# Frictional Characteristics of Deep-Drawing Quality Steel Sheets in the Flat Die Strip Drawing Test

**DOI:** 10.3390/ma15155236

**Published:** 2022-07-28

**Authors:** Marek Szewczyk, Krzysztof Szwajka, Tomasz Trzepieciński

**Affiliations:** 1 Department of Integrated Design and Tribology Systems, Faculty of Mechanics and Technology, Rzeszow University of Technology, ul. Kwiatkowskiego 4, 37-450 Stalowa Wola, Poland; m.szewczyk@prz.edu.pl (M.S.); kszwajka@prz.edu.pl (K.S.); 2 Department of Manufacturing and Production Engineering, Rzeszow University of Technology, al. Powst. Warszawy 8, 35-959 Rzeszow, Poland

**Keywords:** coefficient of friction, deep drawing, sheet metal forming, steel sheet

## Abstract

Friction is one of the most important technological phenomena and has a large influence on the flow characteristics of a deformed material. A strip drawing friction test was used to evaluate the friction characteristics of 0.8 mm thick DC04 steel sheets in a sheet forming operation. Friction tests were carried out using a specially designed friction simulator and uniaxial tensile tests were carried out to determine the mechanical properties of the specimens. In addition, measurements of the sheet surface topography were carried out to identify the tribological properties of the specimens. The friction tests were conducted under different pressure and lubrication conditions. A comparative analysis of the results of the friction tests revealed different changes in the surface topography of the test sheets which can be associated with specific friction mechanisms. It was found that the effectiveness of lubrication depends on the lubricant viscosity and nominal pressure. Increasing the nominal pressure intensifies the phenomenon of asperity flattening and reduces the volume of closed pockets of lubricant.

## 1. Introduction

Friction and wear is an indispensable physical phenomenon accompanying the movement of two bodies in relation to each other. Holmberg and Erdemir [1] found that ~23% of world energy consumption comes from tribological contacts. It commonly assumed that 20% of this energy is used to overcome friction, and 3% is the cost of regenerating worn parts due to failure or wear. The phenomenon of friction occurring in the processes of sheet metal forming (SMF) differs significantly from the phenomena occurring at low loads and in the kinematic nodes of machines. Strong plastic deformation and high temperatures can intensify many tribological phenomena in the contact zone [2,3]. The amount of frictional resistance depends on the surface roughness of the contacting surfaces and on the contact pressures, the value of which may significantly exceed the yield stress of the deformed material [4]. Friction is a phenomenon that has a key impact on the flow of the sheet metal [5,6], the surface finish of the product [7], and also directly affects the wear of tools [8].

Friction in SMF is a complex function of the physical and mechanical properties of the tool and workpiece, process parameters, contact conditions (type of lubricant, oil viscosity) and surface topography of the workpiece and tool [9,10]. Deep drawing is one of the most important technologies, particularly in the engineering, automotive and aircraft industries. During SMF, the relative frictional movement of the tool against sheet metal changes the mechanical properties of the deformed material (e.g., hardness, directional topography and surface roughness) and requires specific properties of the tool surface (e.g., texture, protective coating, thermal-mechanical properties) [11,12]. In order to optimally design the SMF process and control tribological phenomena, it is necessary to understand the interactions between the tribological and surface engineering aspects [13,14]. In order to fully describe the role of friction in sheet metal forming processes, it is necessary to understand the mutual interactions of the tribological system [15], which consists of four main elements—the technological and mechanical parameters of the deformed material and the tool, the lubricant properties, the forming parameters and the characteristics of the tool material [16,17,18].

The strip drawing test is used to model frictional conditions in the flange area of the drawpiece. In this test, the surfaces of the countersamples representing the tool surface are pressed against the sheet metal strip, which is simultaneously drawn [19]. The sheet metal strip is pulled between non-rotating countersamples, which are flat [20] or have a cylindrical shape [19,21]. The possibility of direct measurement of the friction (tangential) and normal force allows high accuracy to be achieved when measuring the value of the coefficient of friction (COF). A change of friction conditions is produced by changing the sliding speed, normal pressure, shape and radius of the countersamples, surface roughness of the countersamples and lubrication conditions [22,23].

The strip drawing test is the most common method of testing the COF and wear of sheet metals. Severo et al. [24] tested the tribological behaviour of W-Ti-N coatings in strip drawing tests. For industrially deposited coatings, a lower COF was measured in dry friction tests for low loads. Tavares et al. [25] studied the friction of commercially coated forming tools. The strip drawing tests showed that the CrN/TiN (HV0.2 = 354 kgf·mm^2^) and VN (HV0.2 = 229 kgf·mm^2^) coatings presented lower and more stable COF values, leading to more favourable tribological conditions. Schell et al. [26] used the strip drawing test to study the influence of the wear performance and friction of different lubricants on a die surface. The results revealed that oil and solid lubricants show distinct behaviour characteristics in the high temperature forming of aluminium sheets. The results from the strip drawing tests conducted by Sutcliffe et al. [27] show a significant effect of the lubrication regime while the properties of the different die materials are less important. Trzepieciński and Fejkiel [19] tested the COF of pre-strained deep drawing quality steel sheets in the strip drawing test with cylindrical countersamples. They concluded that the COF of samples decreases with increasing nominal pressure of the countersamples under both dry and lubricated conditions (machine oil). Trzepieciński [22] tested 0.8 mm thick DC04 steel sheets between two non-rotating countersamples with radii of 10 and 200 mm in lubricated conditions using lubricants based on a combination of boric acid and bio-based oils. The high viscosity of heavy-draw oil (1157 mm^2^·s^−1^) suggested that for low pressures it creates conditions for the formation of a stable lubricant film completely separating the friction surfaces. In another paper, Bazan and Trzepieciński [2] investigated the influence of the surface parameters of the strip specimens, surface parameters of the countersamples and nominal pressure on the COF in the strip drawing friction test. In lubricated conditions with machine oil, the decrease in value of the kurtosis Sku parameter was greater than in dry friction conditions. The lubricated conditions also caused a smaller reduction in the skewness of the Ssk surface roughness parameter than for dry friction conditions.

The aim of this paper is to study of the effect of lubrication conditions and nominal pressure on the frictional properties of low-carbon DC04 steel sheets in the sheet metal–blankholder interface. Commonly available oils were used as lubricants. These oils are relatively cheap compared to professional deep-drawing lubricants used in sheet metal forming. Friction analysis using the strip drawing test is mainly carried out on cylindrical or rounded surface specimens. However, there are a limited number of papers on friction testing using the flat die strip drawing test. Flat countersamples are closer to a real-life model of the friction conditions of the contact properties in the sheet metal–blankholder interface. Therefore, in this article, it was decided to present the results of friction tests of DC04 low-carbon steel sheets with the use of a specially designed simulator which allows the COF to be determined when pulling a sheet strip between flat countersamples. Measurements of the sheet surface topography have been carried out to describe the tribological properties of the specimens. The friction tests have been conducted under different nominal pressure and lubrication conditions in order to find the effect of these parameters on lubrication efficiency and the change in the surface roughness parameters of the specimen surface under contact geometry reflecting friction in the blankholder zone in a conventional deep-drawing process.

## 2. Material and Methods

### 2.1. Test Material

Strip samples for the friction test, approximately 130 mm long and 20 mm wide, were cut along the rolling direction from DC04 (thickness g = 0.8 mm) sheet metal. DC04 (1.0338) is a grade of low-carbon cold-rolled deep-drawing plate mainly intended for use in the automotive industry. The quality requirements for the chemical composition of DC04 steel in accordance with EN 10130:2006 [28] are presented in Table 1.

**Figure 1:**
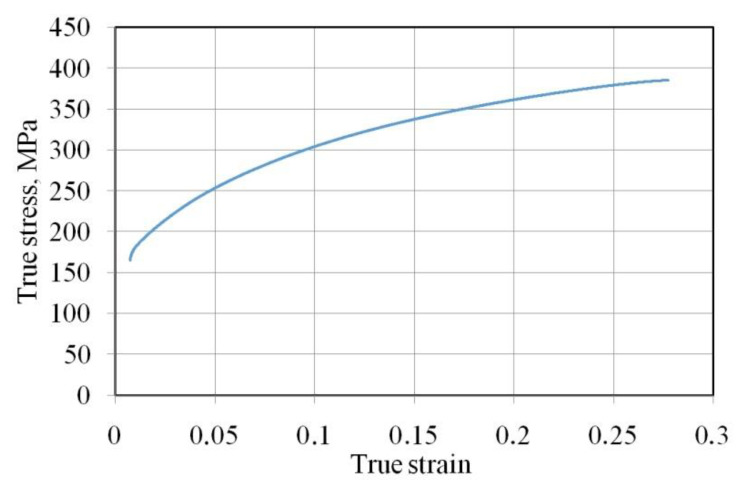
True stress vs. true strain curves for the DC04 steel sheet.

The values of the basic mechanical parameters of the test sheets (Table 2) were determined in a uniaxial tensile test in accordance with ISO 6892-1: 2020 [29]. The samples cut along the rolling direction of the sheet were subjected to uniaxial stretching. Based on the results of the tensile test conducted with three repetitions, the mean values of the following parameters were determined: yield stress R_p0.2_, ultimate tensile strength R_m_, strain hardening coefficient K, and strain hardening exponent n, elongation A_50_ and percentage reduction of area Z. The relation of the true plastic strain and yield stress of DC04 sheets (Figure 1) is exponential and described by the Hollomon equation σ = K·ε^n^.

**Figure 2:**
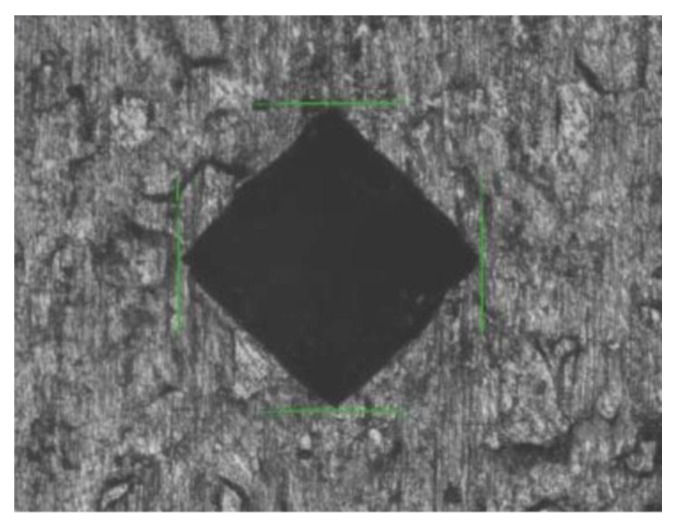
Test point image.

The hardness of the as-received specimens was tested using the Vickers hardness tester series Qness 60 EVO at a test force of 49.03 N. The average hardness was found to be 97 HV5. A view of the test point image is shown in Figure 2.

**Figure 3:**
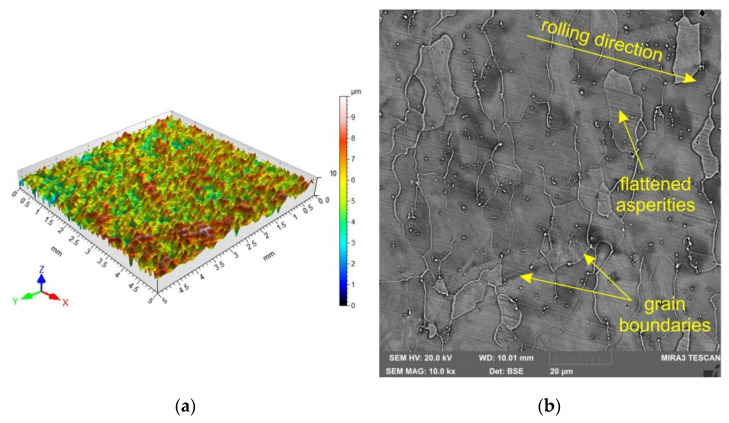
(**a**) Topography of the DC04 sheet and (**b**) SEM micrograph of the in the as-received state.

The topography of the DC04 sheet in the as-received state is shown in Figure 3a. The surface topography of the sheet before and after the friction process was measured with a Hommel-Etamic T8000RC stationary profilometer in accordance with the requirements of the ISO 25178 standard. The basic height parameters of the geometric structure of the surface were determined: arithmetical mean height Sa, skewness Ssk, kurtosis Sku, maximum profile peak height Sp, maximum profile valley depth Sv, maximum height Sz and root mean square deviation Sq. Surface roughness parameters of the as-received surface are as follows: Sa = 1.55 µm, Ssk = −0.0307, Sku = 2.68, Sp = 8.02 µm, Sv = 6.93 µm, Sz = 15.0 µm and Sa = 1.26 µm. The morphologies of the sheet surfaces subjected to friction were examined using a MIRA 3 TESCAN scanning electron microscope (SEM). The MIRA 3 Tescan microscope with a Schottky field emission gun was equipped with a four-quadrant BSE detector. The morphology of the as-received sheet surface is shown in Figure 3b. The surfaces of as-received sheet metals have a granular structure resulting from the sheet manufacturing process (sheet rolling).

### 2.2. The Friction Test Process

**Figure 4:**
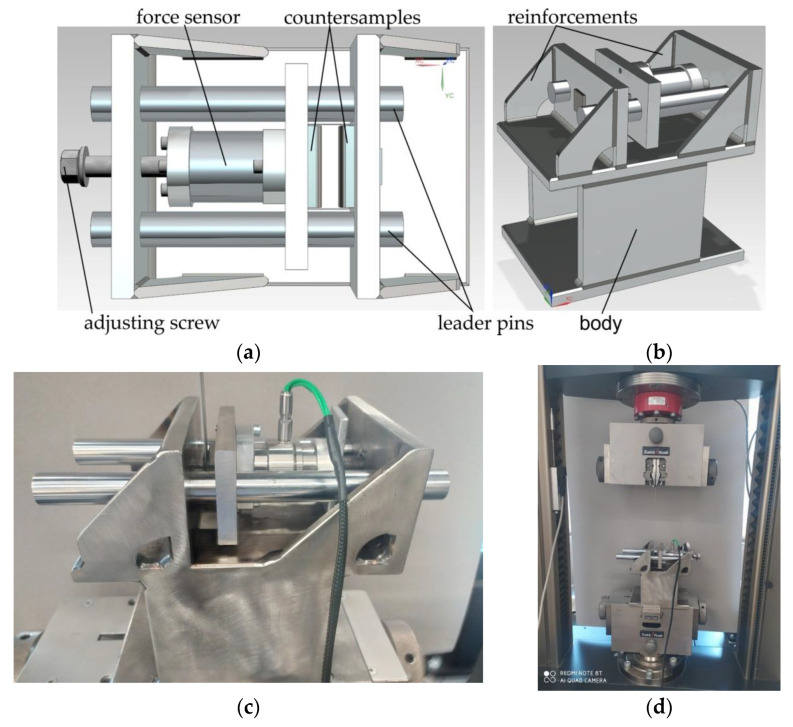
Geometric model of the friction tester in (**a**) top and (**b**) isometric views, (**c**) an actual photo and (**d**) friction tester mounted in Zwick/Roell Z100 testing machine.

The strip drawing test consists in pulling a sheet metal strip between two countersamples with a flat working surface. The model and view of the specially designed tester are shown in Figure 4. The presence of frictional forces on the two contact surfaces is conducive to obtaining greater accuracy of the average value of the COF. The device has the advantage that it is a simple structure with an uncomplicated measurement of the force parameters during the test.

The body of the apparatus was mounted in the lower holder of a Zwick/Roell Z100 testing machine. After the sample was placed between the countersamples, the upper free end of the sample was clamped in the upper holder of the testing machine. The technical parameters of the Zwick/Roell Z100 (Zwick/Roell, Ulm, Germany) testing machine are as follows: maximum testing force −100 kN, traverse speed between 0.0005 and 600 mm/min, accuracy of set rate 0.003% V_nom_, force measurement accuracy with load cell-from 0.2 KN class 1 (from 1 kN class 0.5), resolution of crosshead travel 0.0083 µm/impuls and position accuracy ±2 µm.

The value of the clamping force of the countersamples was set in static conditions (before starting the sample movement) with the help of the adjusting screw (Figure 3a). The normal (clamp) force was measured with a Kistler type 9345B force sensor with a measuring range ±10 kN. Its high resolution allows measurement of the slightest dynamic changes in large forces and torques in non-rotating elements. LabVIEW 2016 software (National Instruments, Austin, Texas, USA) was used to record the clamping force of the countersamples. The value of the tangential (friction) force was measured using the measuring system of the Zwick/Roell Z100 testing machine. Values of both friction and normal forces were correlated in the LabVIEW program based on a Megatron Series SPR18 potentiometric linear transducer.

The samples in the form of sheet strips were cut from the same piece of metal sheet. The strips were tested in as-delivered surface condition [22,23,30]. In general, during friction tests related to sheet metal forming it is assumed [19,22,23,30,31,32,33,34] that samples cut from the same piece of metal sheet have the same surface roughness. These conditions correspond to industrial sheet metal forming, where the surface topography of the workpiece is not strictly the same when forming a series of products.

The sample was pulled through the countersample set with a constant travel speed of the top grip of the testing machine of 2 mm/s. The tests were carried out with the following nominal pressures p 3, 6, 9, 12 MPa. The values of these pressures are within the range of pressures prevailing in sheet metal forming [35,36]. The average value of the COF was determined according to dependence (1), disregarding the transient range r_1_ + r_2_ (Figure 4) related to the change of static friction conditions into kinematic friction.

(1) $$\mu =\frac{F_{T}}{2F_{N}}$$

where: F_T_—friction (pulling) force, F_N_—normal (clamping) force.

**Figure 5:**
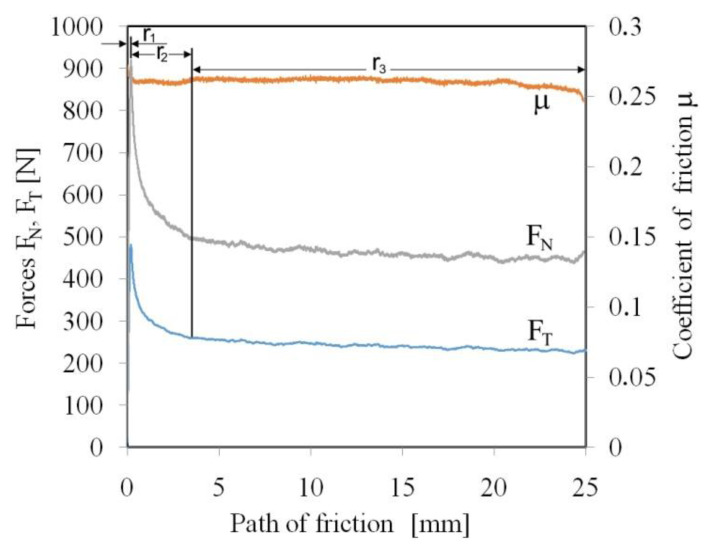
Variation of the friction force, normal force and coefficient of the friction during friction test (dry friction, P = 3 MPa).

**Figure 6:**
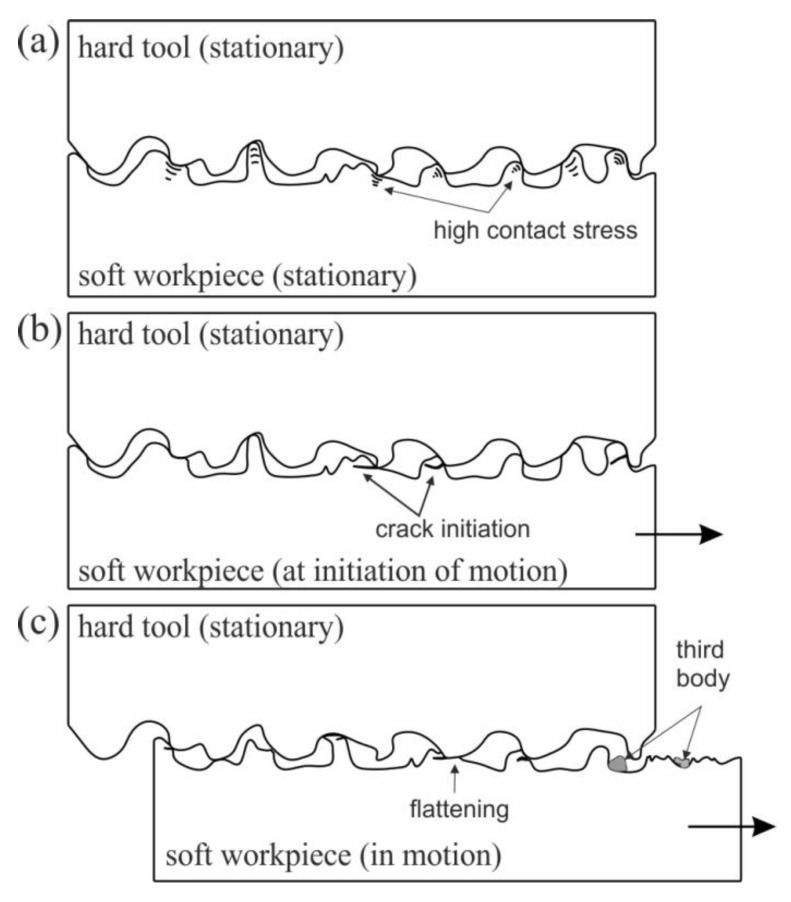
Character of the contact between the surfaces of the hard tool and the soft workpiece: (**a**) no movement, (**b**) initiation of motion and (**c**) in motion.

In the first range (r_1_ in Figure 5), plastic deformation of the highest peaks of the asperities occurs with the formation of large stresses in the material (Figure 6a). At the moment of starting the movement, the deformation of the summits of the asperities increases. At the same time, as a result of the strain hardening phenomenon, their mechanical strength is significantly increased. In such conditions, when a certain stress value is exceeded, the material cracks (Figure 6b). The cracking of the material releases some of the energy accumulated in the material, as a result of which the nominal pressure decreases (the broken particles fill the spaces in the valleys of the asperities (Figure 6c). As a result, the coefficient of friction decreases (r_2_ in Figure 5). In the area of stabilised friction, all asperities of the sheet surface along the length of the contact path with the countersamples are subjected to the flattening mechanism. In the stabilised range r_3_ of the COF (Figure 5), the shear of the summits of asperities occurs at the entrance to the countersample zone. The mean value of the COF was determined for the stabilised range r_3_.

Apart from specialist greases intended for plastic working, many greases and oils manufactured with the intention of being used in mechanical transmissions, engines and hydraulic systems are commonly used in sheet metal forming processes to reduce the value of frictional resistance [37]. The tests were carried out under dry friction conditions (in the as-received sheet metal state) and during lubrication with Castrol Axle EPX 80W-90 gear oil and Castrol EDGE 5W-30 engine oil. EPX 80W-90 is a multi-purpose axle oil which may be used in differentials, final drives and other applications in passenger cars and commercial vehicles. Castrol EDGE with Fluid Strength Technology TM 5W30 oil is a fully synthetic engine oil used in conditions requiring lower viscosity and stronger oils for longer oil change intervals. The criteria for selecting the oils were their general availability and relatively low cost. The viscosity grade of these oils is standardised according to the Society of Automotive Engineers (SAE) and many manufacturers produce oils with the same physico-chemical characteristics. The oils used are generally available and several times cheaper than professional oils intended for deep-drawing operations. Previous investigations on friction of titanium alloy sheets [38] confirmed the effectiveness of typical engine and gear oils in reducing the COF.

The lubrication procedure complies with the guidelines of Payen et al. [39]: apply oil with a soft brush, regularise the deposit with the brush emptied of excess oil (i.e., no drop forms at the end of hairs if weighting brush downward), let the sheet strip stand up for approximately 10 min so that the oil layer homogenises and the excess oil flows away by gravity. The drawing speed was 0.5 mm/s. The basic physical and chemical properties of the oils used in the tests are presented in Table 3.

**Figure 7:**
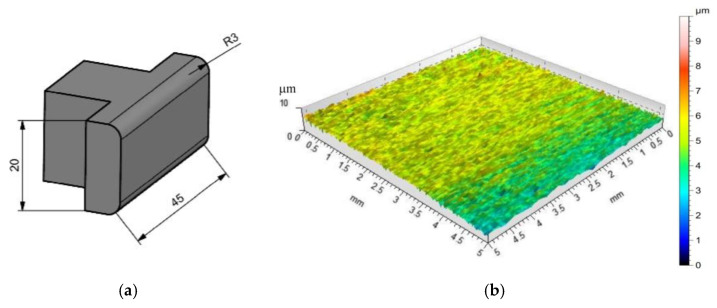
(**a**) Shape and dimensions of the countersamples (units in mm) and (**b**) topography of the countersamples.

Countersamples in the form of inserts (Figure 7) with dimensions of 45 mm wide and 20 mm high incorporated in the construction of the device were made of 145Cr6 (1.2063) cold-worked tool steel. The 145Cr6 steel is recommended for use in forming tools, including stamping dies, punching dies, wire drawing dies, cutting plates and shear knives. The countersamples were fabricated to an overall flatness tolerance of 10 μm. The average hardness was found to be 215 HV10. Twelve measurements were used to calculate the average value of hardness. The topography of the countersamples and basic surface roughness parameters are shown in Figure 7b and Table 4, respectively.

## 3. Results and Discussion

**Figure 8:**
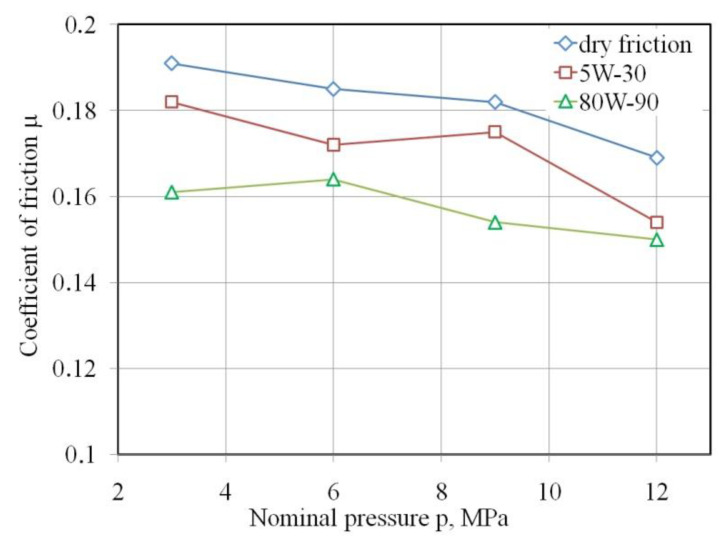
Effect of nominal pressure on the value of coefficient of friction.

There was a trend of a decreasing value of the COF with increasing pressure which was related to the nominal contact surface area (Figure 8). This relationship applies to all the friction conditions analysed. This may be a result of the dependence of friction on the clamping (normal) force, where beyond a certain load the relationship between friction force and clamping force is nonlinear and the value of the COF is not constant. In other words, it changes with normal load. This relation was also observed by Kirkhorn et al. [40], Trzepiecinski et al. [19,41] and Vollertsen and Hu [42]. Friction occurring at high pressure values may significantly differ from the phenomena occurring at low loads and in the kinematic nodes of machines due to the large degree of plastic deformation that can intensify many frictional phenomena in the contact zone. When compared with conventional kinematic machine nodes, the material strength of one element of the friction pair (tool) is assumed to be much greater than the strength of a formed material that undergoes intentional plastic deformation in the sheet metal forming process. The initial topography of the sheet surface under the influence of large deformations is constantly evolving during SMF.

As confirmed by Kirkhorn et al. [40], ten Thije et al. [43], Trzepieciński and Fejkiel [19] and Vollertsen and Hu [42], under such conditions the COF (as a coefficient of proportionality between the friction force and the normal force) has no constant value. In other words, it changes with normal load. It is obvious that the real contact area increases with increasing nominal load due to the intensification of the asperity flattening mechanism. However, determination of the real contact area in sheet metal forming is not fully understood and, due to the random nature of asperities, difficult to precisely determine experimentally. For this reason, the literature on friction testing in SMF is focused on the analysis of nominal pressure.

To determine the influence of the lubricant on the change of friction conditions, an effectiveness of lubrication coefficient W_L_ was introduced as the ratio between the COF determined for dry friction µ_dry_ and that for lubricated conditions µ_oil_ [19]:

(2) $$W_{L}=\frac{\mu_{\mathrm{dry}}-\mu_{\mathrm{oil}}}{\mu_{\mathrm{dry}}}\cdot 100\%$$

**Figure 9:**
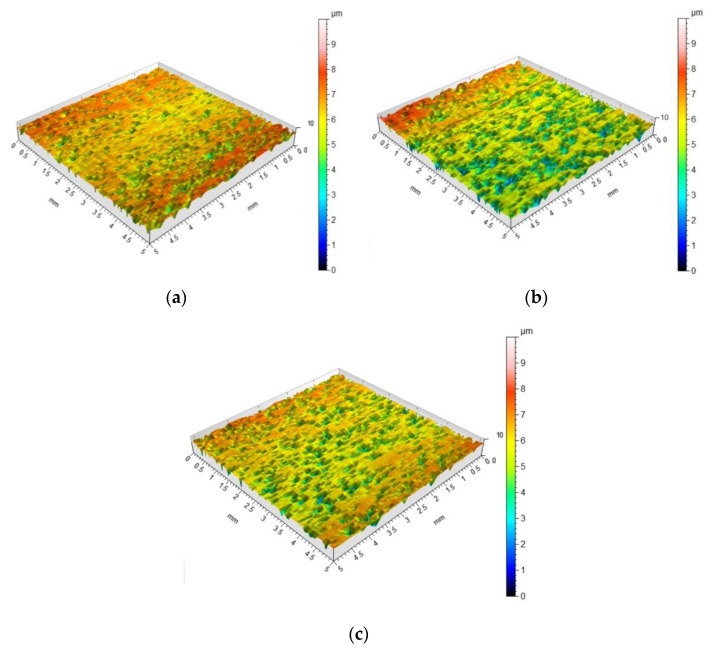
Surface topography of the specimens after a friction test at a nominal pressure of 6 MPa in (**a**) dry friction conditions (Sa = 0.876 µm, Ssk = −1.43, Sku = 5.56, Sp = 2.04 µm, Sv = 6.66 µm, Sz = 8.70 µm, Sa = 0.635 µm), and with (**b**) 5W-30 engine oil (Sa = 1.27 µm, Ssk = −0.698, Sku = 3.65, Sp = 3.47 µm, Sv = 5.38 µm, Sz = 8.85 µm, Sa = 0.965 µm) and (**c**) 80W-90 gear oil (Sa = 1.05 µm, Ssk = −1.36, Sku = 4.78, Sp = 2.46 µm, Sv = 5.88 µm, Sz = 8.34 µm, Sa = 0.782 µm).

During the plastic working of metals, initially there is a small and effective contact area. In elastic contact the surfaces only stick to each other with the summits of the asperities. As the load is increased, there is an increase in the real contact area. The roughness asperities are plastically deformed until the contact surface created in this way is sufficient to transfer the load [44,45]. For smooth surfaces, adhesion is the dominant tribological phenomenon occurring during metallic contact. The asperities of the surface are flattened and sheared which increases the real contact area. A clear flattening of the surface asperities was observed for all analysed friction conditions (Figure 9a–c). It is obvious that the increase in pressure resulted in a more intensive flattening of the asperities. As the surface roughness increases, the influence of adhesion decreases, while the share of the shear mechanism of the sheet metal surface increases.

**Figure 10:**
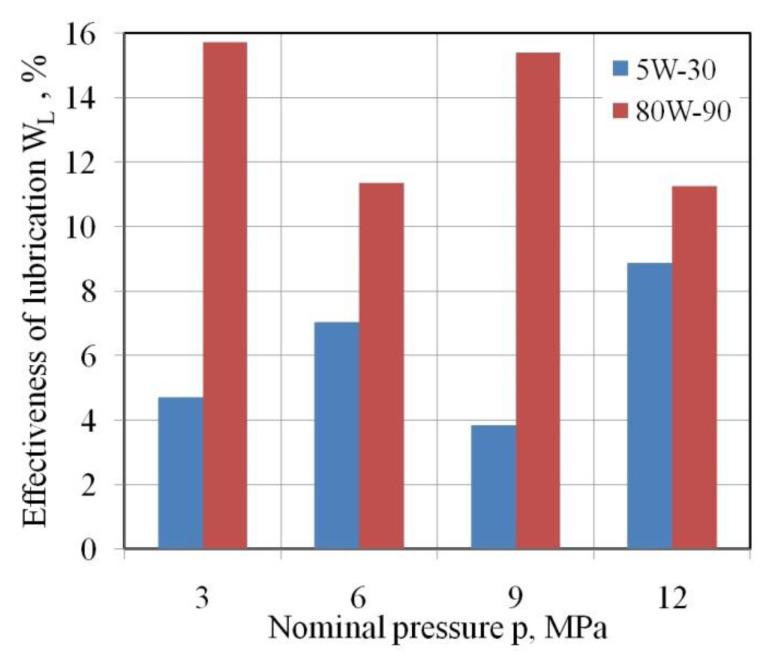
Effect of nominal pressure on the effectiveness of lubrication.

The COF was in the range between 11.24% and 15.7% (Figure 10). With 5W-30 engine oil, which has a kinematic viscosity lower by about 50% compared to gear oil, there is a lower ability to reduce frictional resistance (3.84–8.87%). The effectiveness of lubrication depends on two basic factors: the viscosity of the lubricant and the value of the pressure acting on the surface, which determines the real area of contact. At low pressure, the dominant friction mechanism is the mechanical engagement of the summits of the asperities, and at the same time there are open pockets of lubricant which are not able to generate the appropriate pressure of lubricant separating the mechanical cooperation of the contact surfaces. Under these conditions, a lubricant that requires high viscosity plays a key role. With further increase in nominal pressure, the lubrication efficiency decreases (at 6 MPa for 80W-90 gear oil and at 9 MPa for 5W-30 engine oil). We can discuss a certain optimal pressure, which is a kind of balance between the mechanism of mechanical interaction of the summits of the asperities, the adhesion mechanism, and the volume of closed lubricant pockets generating sufficient hydrostatic pressure to create boundary lubrication. Such favourable conditions can be observed for a pressure of 9 MPa, for lubrication with 5W-30 engine oil. With a further increase in pressure, despite the favourable conditions for creating a “lubricant pad” separating the rubbing surfaces, the real contact area is so large that the mechanism for the flattening of the relatively soft sheet material by the tool material of much greater hardness is intensified. As a consequence, the value of the COF increases for lubrication with 5W-30 engine oil.

Figure 9 adds to the understanding of Figure 7, i.e., that the effectiveness of lubrication, W_L_, increases as the nominal pressure increases, when the lubricant is less viscous (oil 5W-30). Conversely, W_L_ decreases as the nominal pressure increases, when the lubricant is more viscous (oil 80W-90) although the average value of W_L_ is still greater. There are oscillations in W_L_, but they are probably attributable to experimental errors. If the experiment were repeated sufficient times such that the size of the error bars would be clear, then the oscillations would be wiped out, or highlighted.

The pressure–viscosity coefficient α refers to the relationship between the load placed on the oil film (pressure) and the thickness of the oil film (viscosity) at that load, when all other factors (pressure, sliding speed, geometry, temperature) are constant. The units of the pressure–viscosity coefficient α, are mm^2^/N, which are units of area over force. They derive from the relation between viscosity and pressure, commonly expressed by the Barus equation η_p_ = η_0_e^αp^, where η_p_ is viscosity at pressure p (N/mm^2^), η_0_ is viscosity at atmospheric pressure (N/mm^2^) and α is pressure–viscosity coefficient (mm^2^/N). When a is multiplied by pressure with units N/mm^2^, the units cancel giving the nondimensional exponent of the natural number e. An increase in pressure over a confined lubricant results in increasing the lubricant’s viscosity. Such changes in viscosity under mechanical pressure are also known as piezo-viscous effects. This situation exists in certain regimes such as mixed lubrication. Accordingly, as pressure increases—which for a constant force implies a reduced contact area—so does the viscosity, though the relation is not necessarily linear. Additionally, in the case of granular media, it is known that a viscosity increase is accompanied by an increased friction coefficient.

**Figure 11:**
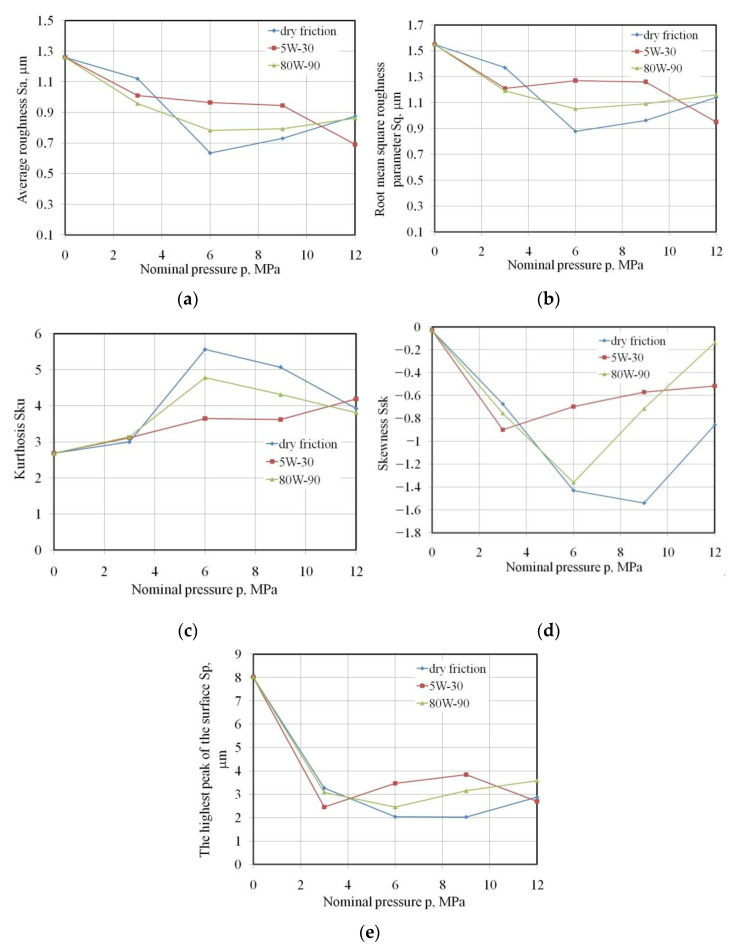
Effect of nominal pressure on variation of the (**a**) Sa, (**b**) Sq, (**c**) Sku, (**d**) Ssk, (**e**) Sp parameters.

Under dry friction conditions, the values of average roughness Sa and root mean square roughness Sq parameters decrease in the pressure range between 3 and 6 MPa (Figure 11a,b) as a result of the flattening mechanism. Increasing the pressure activates the ploughing mechanism involving the mechanical interaction of the highest summits of the tool surface with the flattened surface of the sheet metal. As a result, the COF increases slightly. Under lubricated conditions, the adhesion phenomenon in the areas of real contact practically does not occur, while the flattening and ploughing mechanisms are limited by the hydrodynamic interaction of the lubricating film accumulated in the valleys of the surface topography. When lubricating the sample surfaces with the oils investigated, there is a clear tendency to a reduction of the Sa and Sq parameters with increasing nominal pressure.

For engine oil lubrication conditions, the surface slope coefficient, named kurtosis Sku, increases almost linearly with increasing nominal pressure (Figure 11c). In the remaining two friction conditions analysed, after a sharp increase in this parameter during a change in pressure from 3 to 6 MPa, kurtosis decreases.

A positive value of the surface asymmetry coefficient, named skewness Ssk, reduces the COF, while in the case of negative skewness, the friction is more intense. Hence, the most unfavourable friction conditions related to the skewness parameter occur at pressures of 3 MPa, 6 MPa and 9 MPa, for the lubrication conditions with 5W-30 engine oil, 80W-90 gear oil and lack of lubrication, respectively (Figure 11d). 

The occurrence of the flattening phenomenon during the cooperation of the sheet surface with the countersample surface is evidenced by the reduction of the highest peak of the surface Sp by over 50% in relation to the as-received surface. There is also a certain correlation between the character of changes in the parameters Sq (Figure 11b) and Sp (Figure 11e).

During loading, the summits of the asperities deform elastically and plastically, changing the surface topography. In the case of elastic–plastic metals, the increase in pressure during the forced sliding movement causes an increase in the real contact surface, which may lead to a reduction in the volume of open pockets of lubricant [46]. Closed pockets of lubricant are separated from the outer edges of the material and store the lubricant in the closed volume of the valleys. Under load, the pressure of the lubricant increases. This forms a kind of hydrostatic cushion, which takes over part of the load [46].

**Figure 12:**
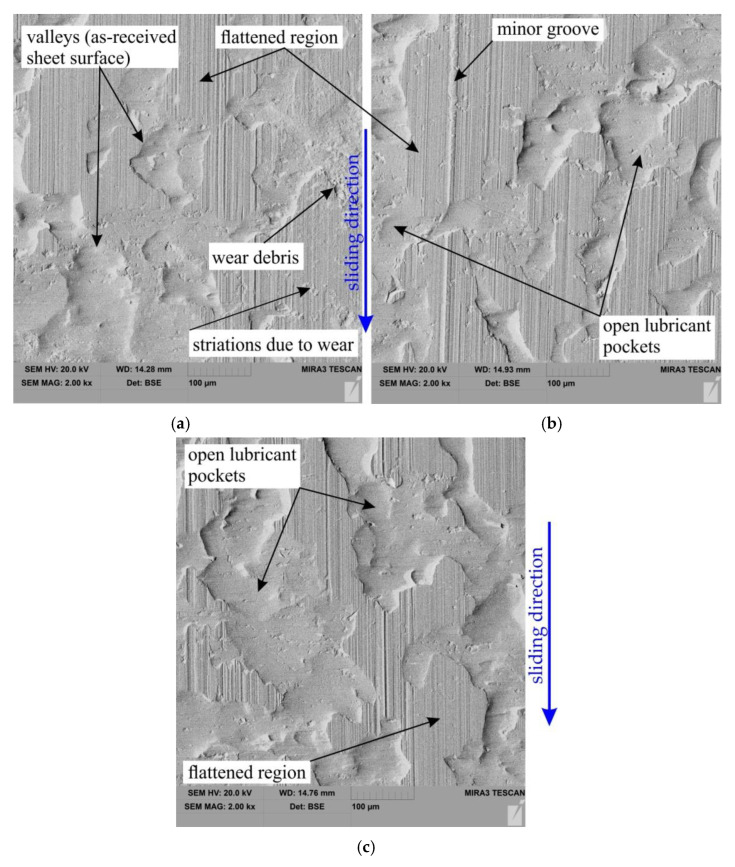
SEM micrographs of the sheet surface after friction tests carried out under the following conditions (nominal pressure P = 3 MPa): (**a**) dry friction, (**b**) 5W-30 oil and (**c**) 80W-90 oil.

**Figure 13:**
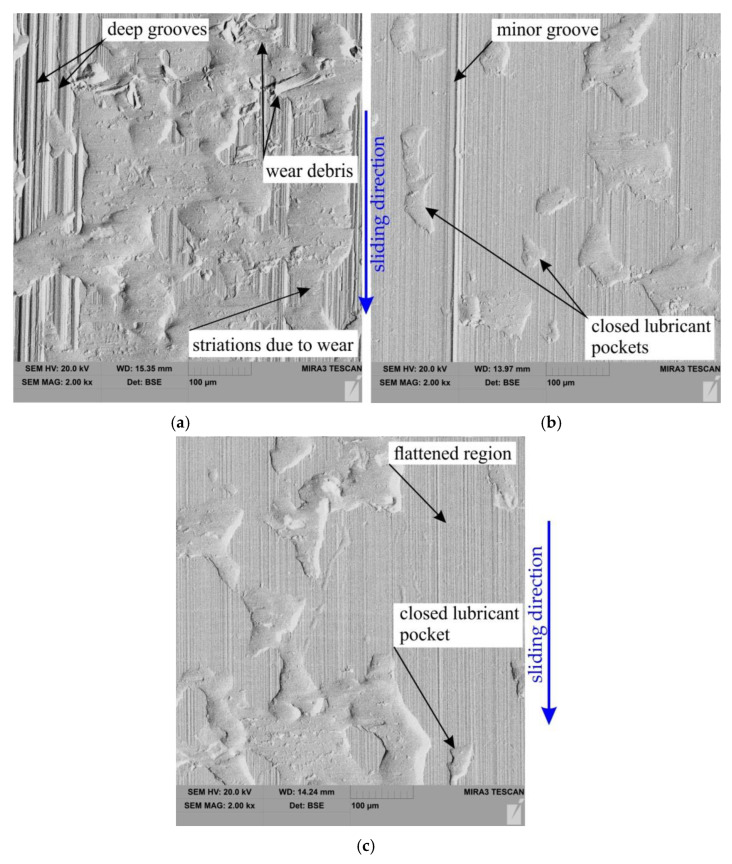
SEM micrographs of the sheet surface after friction tests carried out under the following conditions (nominal pressure P = 12 MPa): (**a**) dry friction, (**b**) 5W-30 oil and (**c**) 80W-90 oil.

SEM micrographs of the sheet surface after friction tests carried out under the nominal pressure P = 3 MPa and P = 12 MPa are shown in Figure 12 and Figure 13, respectively. The summits of the surface were deformed creating flattened areas on the surface of the sheet (Figure 12). Increasing the nominal pressure increases the surface roughness of the sheet with clear traces of flattening. At the same time, in these areas, the impact of the ploughing phenomenon is intensified, especially in dry friction conditions when there are favourable conditions for revealing the scuffing mechanism (Figure 13a).

## 4. Conclusions

This article presents the results of friction tests of low-carbon DC04 steel sheets destined for deep-drawing operations. Based on the strip drawing test that simulates the friction phenomena in the area of the blankholder of the stamping die, the following conclusions can be drawn:

- The value of the COF decreases with increasing nominal pressure for all analysed friction conditions.
- Use of 80W-90 gear oil ensured, depending on the value of the pressure, a reduction in the value of the COF in the range between 11.24% and 15.7% compared to dry friction conditions.
- In the entire range of nominal pressures examined, the engine oil decreased the value of the COF by only 3.84–8.87%.
- In dry friction conditions, the average roughness Sa and the root mean square roughness Sq decrease in the pressure range of 3–6 MPa, and then an increase in these parameters is observed as a result of the intensification of the ploughing mechanism. On the other hand, when lubricating the sample surfaces there was a clear trend to a reduction of the Sa and Sq parameters with increasing nominal pressure in the whole range of pressures investigated.
- The occurrence of the flattening mechanism during cooperation of the relatively soft surface of the sheet metal with the hard surface of the countersamples causes a reduction in the highest peak of the surface Sp by over 50% in relation to the as-received surface.

## Figures and Tables

**Table 1:** Chemical composition of DC04 in accordance with the requirements of EN 10130: 2006 (in wt.%).

C (Max.)	Mn (Max.)	P (Max.)	S (Max.)	Fe
0.08	0.4	0.03	0.03	balance

**Table 2:** Basic mechanical parameters of DC04 steel sheet.

R_p0.2_, MPa	R_m_, Mpa	A_50_, %	Z, %	K, Mpa	n
165.2	309.3	37.1	45.7	526.9	0.23

**Table 3:** Basic physico-chemical properties of the oils.

Oil	Property	Test Method	Value
Castrol Axle EPX 80W-90	Density at 15 °C	ASTM D4052	0.896 g/mL
	Kinematic viscosity at 40 °C	ASTM D445	134 mm^2^/s
	Viscosity index	ASTM D2270	101
	Pour point	ASTM D97	−33 °C
	Flash point	ASTM D92	222 °C
Castrol EDGE 5W-30	Density at 15 °C	ASTM D4052	0.851 g/ml
	Kinematic viscosity at 40 °C	ASTM D445	70 mm^2^/s
	Viscosity index	ASTM D2270	169
	Pour point	ASTM D97	−42 °C
	Flash point	ASTM D93	202 °C

**Table 4:** Basic surface roughness parameters of countersamples.

Sa, µm	Sq, µm	Sku	Ssk	Sp, µm	Sv, µm	Sz, µm
0.636	0.810	3.76	−0.544	4.89	5.11	10.0

## Data Availability

The data presented in this study are available on request from the corresponding author.
